# 16S rRNA Long-Read Sequencing of the Granulation Tissue from Nonsmokers and Smokers-Severe Chronic Periodontitis Patients

**DOI:** 10.1155/2018/4832912

**Published:** 2018-06-14

**Authors:** Rebecca Chowdhry, Neetu Singh, Dinesh Kumar Sahu, Ratnesh Kumar Tripathi, Archana Mishra, Anjana Singh, Hari Shyam, Pratap Shankar, Nand Lal, Madan Lal Brahma Bhatt, Ravi Kant

**Affiliations:** ^1^Department of Periodontology, King George's Medical University, Lucknow 226 003, India; ^2^Molecular Biology Unit, Center for Advance Research, King George's Medical University, Lucknow 226 003, India; ^3^Imperial Life Sciences, 463 Phase City 2nd, Sector 37, Gurgaon, Haryana 122001, India; ^4^Department of Thoracic Surgery, King George's Medical University, Lucknow 226 003, India; ^5^Department of Pulmonary and Critical Care Medicine, King George's Medical University, Lucknow 226 003, India; ^6^King George's Medical University, Lucknow 226 003, India; ^7^All India Institute of Medical Sciences, Rishikesh 249 203, India

## Abstract

Smoking has been associated with increased risk of periodontitis. The aim of the present study was to compare the periodontal disease severity among smokers and nonsmokers which may help in better understanding of predisposition to this chronic inflammation mediated diseases. We selected deep-seated infected granulation tissue removed during periodontal flap surgery procedures for identification and differential abundance of residential bacterial species among smokers and nonsmokers through long-read sequencing technology targeting full-length 16S rRNA gene. A total of 8 phyla were identified among which* Firmicutes *and* Bacteroidetes *were most dominating. Differential abundance analysis of OTUs through PICRUST showed significant (p>0.05) abundance of Phyla-Fusobacteria (*Streptobacillus moniliformis*); Phyla-Firmicutes (*Streptococcus equi*), and Phyla Proteobacteria (*Enhydrobacter aerosaccus) *in nonsmokers compared to smokers. The differential abundance of oral metagenomes in smokers showed significant enrichment of host genes modulating pathways involving primary immunodeficiency, citrate cycle, streptomycin biosynthesis, vitamin B6 metabolism, butanoate metabolism, glycine, serine, and threonine metabolism pathways. While thiamine metabolism, amino acid metabolism, homologous recombination, epithelial cell signaling, aminoacyl-tRNA biosynthesis, phosphonate/phosphinate metabolism, polycyclic aromatic hydrocarbon degradation, synthesis and degradation of ketone bodies, translation factors, Ascorbate and aldarate metabolism, and DNA replication pathways were significantly enriched in nonsmokers, modulation of these pathways in oral cavities due to differential enrichment of metagenomes in smokers may lead to an increased susceptibility to infections and/or higher formation of DNA adducts, which may increase the risk of carcinogenesis.

## 1. Introduction

Smoking continues to be the leading cause of chronic inflammations leading to cancer, despite the extensive knowledge that smoking and tobacco products are injurious to health. Chronic Periodontitis (CP) is a type of inflammatory disease of the supporting tissues of the teeth, which results in loss of connective tissue attachment, alveolar bone, and, ultimately, loss of the teeth [1]. The initiation and progression of periodontitis are modulated by genetic susceptibility of the host and lifestyle factors [2]. Among these, smoking and tobacco products seem to be the most important one [3]. Smokers show higher prevalence and severity of periodontal destruction as is evident through significant tendency of greater probing depth and clinical attachment level means; greater amount of plaque in all regions; greater gingival index means and the therapy of periodontitis in smokers on average are less effective [3–5]. As the time of smoking increases, it further complicates the diseases by affecting the immune response of the host [6]. Smokers with periodontitis show lesser serum antibodies in particular immunoglobulin G class 2 (IgG2) and impaired function of leucocytes [7–9]. It has been also hypothesized that smoking can affect the composition of the subgingival microbiome [10]. However, earlier investigations using targeted approaches (techniques focused on detection of specific microorganisms) have shown contradictory results. On one side, studies reported that smokers with periodontitis show higher prevalence and quantity of the traditional periodontitis-associated pathogens in comparison with nonsmokers [11–13], while on the other side investigations could not confirm those results [14–16]. Discrepancies are understandable as the subgingival microbiome is complex and based on the interaction of a large number of bacterial taxa, whose major part remains uncultivated [17, 18]. Further, the advent of high-end techniques to investigate the complete microbiome has provided a better understanding of the complex oral microbiological environment between smokers and nonsmokers. In earlier study the microbiota of smokers and nonsmokers was compared through terminal restriction fragment length polymorphism and high-throughput sequencing approaches and have shown changes in higher proportion of* Firmicutes *and* Actinobacteria *and a lower proportion of* Bacteroidetes *and* Proteobacteria *in the intestinal microbiota [19] and fecal material in humans and correlated these results with metabolic effects [20, 21].

Different sequencing methods have been used to explore the microbial complexity of CP samples where most of the techniques used a short variable region for sequencing. Most recent publications showed that long-read sequencing technology (V1-V9 full-length variable region of a 16S rRNA gene) can provide finer phylogenetic profiling [22]. The aim of this study was to compare 16S rRNA sequencing through PacBio in a population of patients affected by CP and differentiated on the basis of smoking habit through long-read sequencing technology using V1-V9 primers.

## 2. Materials and Methods

### 2.1. Screening of Patients and Sample Collection

A total of 30 Chronic Periodontitis (CP) patients aged between 35-67 years with a median age of 51 years were screened. Five patients with average age of 53.2 years (CP_B1, CP_B2, CP_C1, CP_C2, and CP_D1) with the history of smoking and tobacco chewing, bleeding on probing, Periodontal pockets ≥ 5 mm and having Clinical attachment loss (CAL) were selected as an experimental group (CP+S). Five patients of corresponding age group (CP_6, CP_7, CP_8, CP_15, and CP_20) with the history of no smoking and tobacco chewing, bleeding on probing, periodontal pockets ≥ 5 mm and having Clinical attachment loss (CAL) were included as a control (CP**) (**Supplementary Table 1**). **All the samples were obtained after informed consent and the study was approved by the Institutional Ethics committee, King George's Medical University. All the experiments have been performed in accordance with relevant guidelines and regulation. Both the groups were also screened for the history of systemic disorders (diabetes, hypertension, etc.), blood dyscrasias, which compromised the immune system, and consumption of antibiotics for the past three to six months. Clinically, patients were evaluated for diagnostic parameters like oral hygiene index; plaque index; gingival bleeding index; periodontal pockets examination, clinical attachment level (CAL), furcation level; radiographic evaluation: OPG or IOPA. Based on these criteria patients were subjected to routine scaling and root planning. However during treatment, patients were also advised with the cessation of these harmful habits completely, with the instructions of strict oral hygiene for 1 week. Subsequently, periodontal flap surgery was conducted in the categorized patients and granulation tissue was collected during flap surgery in sterile Eppendorf tubes on ice, transported to the laboratory, and stored at -20°C until further processing.

### 2.2. DNA Extraction

Bacterial genomic DNA was isolated from collected granulation tissue using Qiagen mini-DNA isolation Kit, Qiagen, and stored at -20°C till further analysis. The quality and quantity of isolated genomic DNA were performed through Quawell spectrophotometer (Quawell Technology Inc., San Jose), agarose gel electrophoresis, and Qubit Fluorimeter (Agilent, Santa Clara, CA, USA). Genomic DNA having absorption ratio A260/A280 in the range 1.8-2.0 was considered for 16S rRNA gene amplification.

### 2.3. 16S rRNA Gene Amplification, Sample Barcoding, and PacBio Sequencing

A total of 50 ng of genomic DNA from 5 CP (control) and 5 CP+S (experimental) patient groups was used for the 16S rRNA gene amplification. Polymerase chain reactions (PCR) amplification was performed for each sample with forward and reverse V1-V9 gene-specific primers (Table 1). Briefly, PCR reactions were performed in a reaction volume of 50 *μ*l together with 300 *μ*M dNTPs, 0.3 *μ*M barcoded forward and reverse primers, and 1 U/ *μ*l KAPA HiFi Hot Start DNA Polymerase. Cycling conditions were as follows: denaturation at 95°C for 2 minutes, followed by 27 cycles of amplification (denaturation 95°C for 30 seconds, annealing 57°C for 30 seconds, and extension 72°C for 30 seconds) and a final extension at 72°C for 5 minutes. In the next round of amplification, the 5' ends of the both forward and reverse primers were barcoded with paired 16 bases symmetric barcodes (https://github.com/PacificBiosciences/Bioinformatics-Training/wiki/Barcoding-with-SMRT-Analysis-2.3) for multiplexing of samples within a single sequencing run. Further, the amplicon product (1464 bp, V1-V9 region) was purified with 0.45X AMPure PB beads (Pacific Biosciences, Menlo Park, CA, USA) and checked for the expected size on Caliper LabChip GX (Perkin Elmer, Hopkinton, MA, USA) and further quantified with Qubit fluorometer Quant-iT dsDNA BR Assay Kit (Thermo Fisher Scientific, Waltham, MA, USA).

Equimolar concentrations of purified amplicons from each group were pooled separately and further used for single-molecule real-time (SMRT) bell library preparations was performed following the manufacturer's instructions (PacBio). Briefly, 500 ng of PCR amplified amplicon was used for DNA damage repair, followed by end repair and purification through 0.45X AMPure PB beads and further ligation of blunt-end adaptors to end repaired products. After exonuclease treatment and purification with 0.45X AMPure PB beads, SMRT bell libraries were prepared. A total of 4 SMRT cells (2 SMRT per group) were used for sequencing of barcoded 16S rRNA amplicons SMRT bell libraries, using P6C4 chemistry with 6 hrs collection protocol on PacBio RSII.

### 2.4. Data Analysis

Generated raw sequences were processed through the PacBio SMRT Analysis 2.3.0 using RS_Read of Insert (ROI) algorithm. Sequences were filtered for a minimum of 2 passes and a minimum predicted the accuracy of 95% to filter the reads with high sequencing error rate. The CCS reads were demultiplexed by means of command line interface “pbbarcode” and barcode-FASTA having mix-and-match sets of forward and reverse barcodes, using a minimum barcode score of 23. Sequence files and metadata for all samples used in this study have been deposited in SRA under Bio project; PRJNA451246 and Biosample; SAMN08966100.

High-quality CCS reads of each sample were projected for downstream analysis through Mothur (version 1.34.4) package to species-level identification [23], whereas phyla and genus level classifications were performed through the MG-RAST metagenome analysis web server [24]. For the MG-RAST analysis individual samples, “fasta” files were uploaded and processed through the pipeline which includes multiple steps of quality control, removal of artifacts, identification of ribosomal feature against* SILVA *and* RDP *databases, and identification of taxonomic origin for each feature [24], while for Mothur package analysis selected commands were used for data processing. Initially, the sequences were subjected to amplicon size trimming protocol to remove sequences outside the expected amplicon size (<1400 bp and >1600 bp) and homopolymeric-sequences. Unique sequences produced through above process were aligned against “Greengenes reference database” release green gene gg_13_8_99. The aligned-sequences were screened for alignment outside the expected alignment coordinates, min score to 80 or 90 (minimum alignment score) and the minimum to 80 or 90 (minimum similarity score), which were subsequently removed. The filtered sequences were preclustered allowing 1% mismatch. As a universal rule, we allowed 1% difference (1 bp/100 bp of the 16S rRNA gene) for the bacterial 16S rRNA gene. Sequences were screened for chimeras using Chimera Uchime. High-quality filtered CCS reads sequences were clustered into operational taxonomic units (OTU) using a cutoff of 0.10. High-quality sequences and OTUs were classified using the gg_13_8_99.gg.tax database, while the other databases only provided taxonomic data to the genus level [25, 26]. Representative sequences from each OTU were picked and assigned taxonomy using the classify.seq command. During this process, sequences with high identity (>97%) were grouped into the same OTU and are reported at the species level of taxonomic identification to all sequences, wherever they are reported [23]. Further, unknown and unclassified taxonomic OTU's were removed. Matrices of Alpha (Ace, Chao, shannon, invsimpson, sobs index) and beta diversity (jclass, thetayc, nmds) were generated using Mothur package. Rarefaction curves, principal coordinates (PCoA), and nonmetric multidimensional scaling (NMDS) were generated using PASTv3.11 (http://palaeo-electronica.org/2001_1/past/issue1_01.htm).

### 2.5. Metagenome Function Predictions

Metagenomic functions conclusions from the 16S rRNA data were made using PICRUSt (Phylogenetic Investigation of Communities by Reconstruction of Unobserved States) method utilizing computational based approach for predicting the functional composition of a metagenome based on marker gene data against a reference genomes database [27] and Kyoto Encyclopedia of Genes and Genomes (KEGG) pathways [28]. Statistics and visualization of functional data were depicted using STAMP [29, 30]. Closed-reference OTU-picking protocols were used to identify 16S rRNA sequences belonging to annotated genomes, as described in the section above. Briefly, sequences were grouped into OTUs based on 97% sequence identity using* uclust *and the* Greengenes *reference database. The taxonomy was assigned to representative sequences from each OTU using uclust consensus taxonomy assigner. PICRUSt was used to generate a list of functional genes predicted to be present in the sample and to organize these genes into gene pathways. The OTUs abundance was normalized automatically using 16S rRNA gene copy numbers from known bacterial genomes in Integrated Microbial Genomes (IMG) [31]. The predicted genes and their function were associated with KEGG pathways. Differences between the abundance of functional pathways and species richness among groups (CP and CP+S) were compared through software STAMP (http://kiwi.cs.dal.ca/Software/STAMP) [29, 30]. Heatmaps clustering, chi square, and PCA plots were used to display differences between the groups. Two-side Welch's t-test and Benjamini-Hochberg FDR correction were used in two groups analysis. All analyses were performed with the significance level of p>0.05.

## 3. Results

### 3.1. Characteristics of Samples, Data Generation, and Quality Filtering

A total of 10 samples were analyzed in this study where all subjects were divided into control (5, CP cases; based on criteria described in the Methods section) and experimental (5, CP + Smoking cases) groups according to dental examination results 1 week after surgery was performed** (**Supplementary Table 1). Granulation tissue sample was collected from all subjects and bacterial species richness as well as metagenome functional prediction analysis was performed to compare between control and experimental groups. The raw data contained 166,968,561 read bases with the mean read length of the insert of 1,517 bases; mean read quality of insert of 98.53% and 12 mean numbers of passes. After demultiplexing with respect to individual barcoded samples and filtering out low-quality reads (>85%) and host contamination a total of 1,63,546 posttrimmed circular consensus sequence (CCS) reads with 16,354 reads per sample on average were obtained (Supplementary Table 1). Further, removal of chimeras and CCS reads made shorter than 1400 bases and longer than 1600 bases and a total of 99,166 high-quality CCS reads were obtained (Supplementary Table 1).

### 3.2. Characterization of 16S rRNA Gene at Phylum/Genus/Species Level in Control and Experimental Granulation Tissue Samples Using PacBio Sequencing

The 16S rRNA gene sequences were assigned to individual species-level OTUs at 3% dissimilarity against Greengenes database (gene_13_8_99.gg.tax), while phyla and genus were determined through MG-RAST databases for species richness in control and experimental samples. Phylum level characterizations of 16S rRNA gene with MG-RAST showed among individual experimental (smokers) samples included 10 phyla with 27 genera while 14 phyla with 24 genera were identified among individual control (nonsmokers) samples (Supplementary Figures 1, 2, 3, and 4). Overall, the most abundant (more than 40% representation) phyla comprised* Bacteroides* and* Firmicutes* between both the groups. From the experimental group,* Bacteroides* were most abundant in CP_B1 (65.58%), CP_B2 (57.30%), CP_C2 (59.75%), and CP_D1 (59.72%), whereas in CP_C1* Firmicutes* (64.70%) showed the most abundance (Supplementary Figure 1). However in control group* Bacteroides* (CP_7; 77.95%, CP_20; 55.33%),* Firmicutes* (CP_6; 80.71%), and* Actinobacteria* (CP_8; 44.38%) showed comparatively higher representation (Supplementary Figure 3).

Genus level comparisons showed that from experimental group* Capnocytophaga *(CP_B1; 41.96%),* Porphyromonas *(CP_D1; 45.27%), and* Veillonella *(CP_C2; 73.60%) were dominantly observed (Supplementary Figure 2). Similarly, from the control group* Veillonella *(CP_6; 82.99%),* Porphyromonas *(CP_7; 75.8%), and* Actinomyces *(CP_8; 45.38%) were predominantly observed (Supplementary Figure 4).

The species-level classification of 16S rRNA gene against green gene_13_8_99.gg.tax database was carried out after pooling individually control and experimental samples into two groups through Mothur pipeline. The results showed a total of 8 assigned phyla with 2 of these dominating across all the samples: Firmicutes (57.88%; control and 52.73%; experimental) and Bacteroidetes (22.17%; control and 24.99%; experimental) (Table 2). The occurrence was comparable in both control and experimental groups and similar to those observed with MG-RAST pipeline. Among* Firmicutes*,* Selenomonas noxia *(control; 31.99% and experimental; 40.80%),* Selenomonas bovis *(control; 19. 56% and experimental; 24.80%), and* Veillonella parvula *(control; 19.46% and experimental; 23.94%) showed predominance. In* Bacteroidetes*,* Capnocytophaga ochracea *(control; 55.03% and experimental; 56.49%) and* Macellibacteroides fermentans *(control; 16.95% and experimental; 15.69%) were highly represented. Apart from these two highly abundant taxa,* Proteobacteria *were represented by 11.85% in control and 13.06% in experimental group which comprised* Neisseria oralis *(control; 46.63% and experimental; 44.94%),* Campylobacter rectus *(control; 36.35% and experimental; 37.71%) and* Prevotella nigrescens *(control; 14.21% and experimental; 14.33%). In addition,* Sebaldella termitidis *species from* Fusobacteria* (4.73% control; 5.45% experimental) was present in both control and experimental groups with 99.99% dominance. Also among* Actinobacteria* (control; 2.03% and experimental; 2.33%),* Actinomyces hyovaginalis *(control; 35.60% and experimental; 35.75%),* Rothia terrae *(control; 31.00% and experimental; 31.09%), and* Corynebacterium pilosum *(control; 24.41% and experimental; 24.81%) were dominantly classified.

### 3.3. Species Richness and Rarefaction Curve Analysis

Species richness or *α*-diversity summarizes the diversity of organisms in a sample with a single number. The *α*-diversity of annotated samples can be estimated from the distribution of the species-level classifications. In experimental or CP+S group the *α*-diversity was characterized by the observation of 7 (CP_B1), 36 (CP_B2), 12 (CP_C1), 10 (CP_C2), and 20 species (CP_D1). Similarly, from the control or CP group, *α*-diversity observation showed the presence of 10 (CP_6), 13 (CP_7), 28 (CP_8) 39 (CP_15), and 72 species (CP_20) (Supplementary Figure 5). Further analysis of rarefaction curve of annotated species richness showed a saturation curve in all of the control and experimental groups (Supplementary Figure 6). A steep slope indicates that a large fraction of the species diversity remains to be discovered. Sampling curves generally rise very quickly at first and then level off toward an asymptote as fewer new species are found per unit of individuals collected.

### 3.4. Differential Abundance of the Microbial Community in CP Granulation Tissue Samples between Nonsmokers and Smokers and Associated Functions

**Figure 1:**
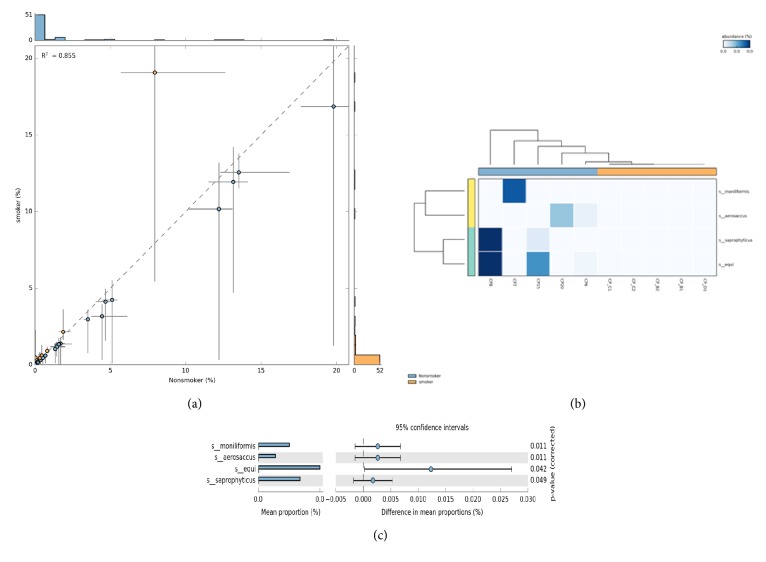
(a)** Scatter plot and correlation estimates** of bacterial species abundance between nonsmokers and smokers in granulation tissue of chronic periodontitis samples. (b) Heatmap profile of abundance distribution of identified species in between nonsmokers and smokers in granulation tissue of chronic periodontitis samples. (c) Mean proportion of bacterial species abundance between nonsmokers and smokers in granulation tissue of chronic periodontitis samples. The significant differences observed between the two groups at 95% confidence level and p>0.05 are reported.

The 16S rRNA gene CCS reads were assigned to species-level OTUs at 3% dissimilarity. In total, 2,956 OTUs were detected in all 10 individuals and were compared between control and experimental samples. OTU based correlation coefficient analysis showed R^2^ =0.855 between experimental and control samples depicting a poor relationship between microbial abundance between the two groups (Figure 1(a)). STAMP based generation of OTU heatmap among nonsmokers and smokers showed that CP_6, CP_7, CP_8, CP_15, and CP_20 were in one cluster while CP_B1, CP_B2, CP_C1, CP_C2, and CP_D1 were in a different cluster (Figure 1(b)). PICRUST analysis of normalized OTU's showed the dominance of Phyla-*Fusobacteria*-*Streptobacillus moniliformis*; Phyla-*Firmicutes*-*Streptococcus equi*; Phyla* Proteobacteria*-*Enhydrobacter aerosaccus*; Phyla-*Firmicutes*-*Staphylococcus saprophyticus *in nonsmokers (p>0.05) compared to smokers (Figure 1(c)).

**Figure 2:**
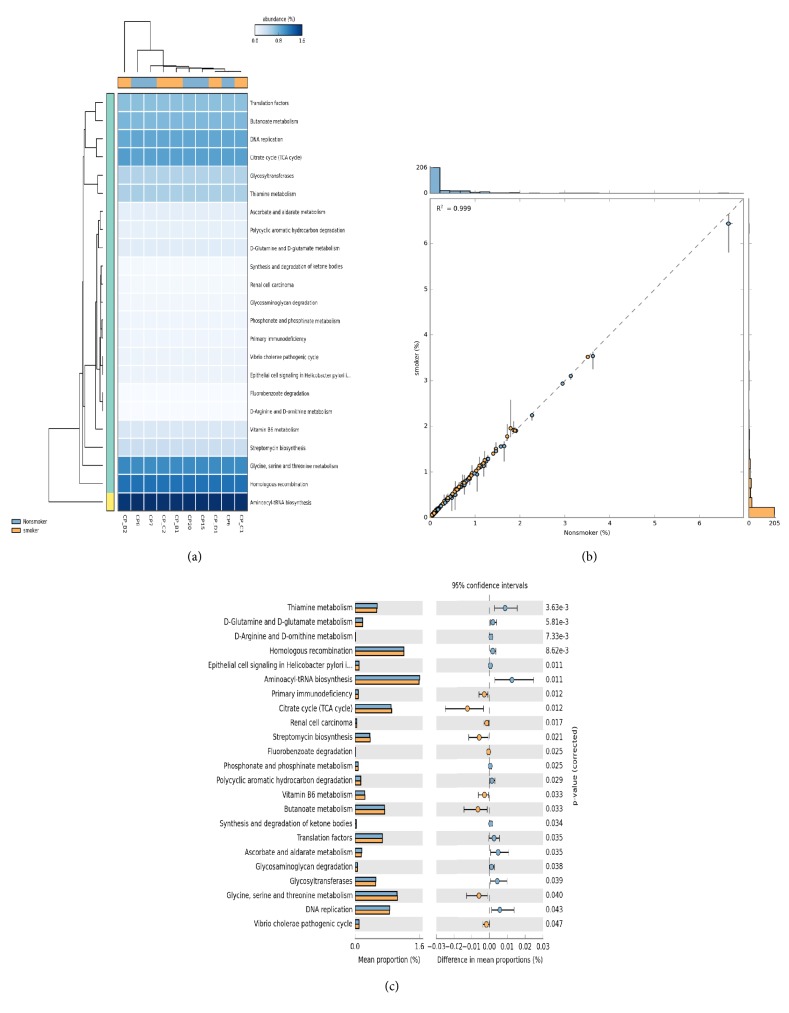
**Heatmap profile of predicted functions** of identified bacterial species between nonsmokers and smokers in granulation tissue of chronic periodontitis samples. Scatter plot and correlation estimates of bacterial species functional predictions between nonsmokers and smokers in granulation tissue of chronic periodontitis samples. Mean proportion of bacterial species functional predictions between nonsmokers and smokers in granulation tissue of chronic periodontitis samples. The significant differences observed between the two groups at 95% confidence level and p>0.05 are reported.

Functional predictions of the observed species abundance showed the formation of no distinct clusters in heatmap profiles (Figure 2(a)) with a correlation coefficient of R^2^ = 0.999 (Figure 2(b)). primary immunodeficiency, citrate cycle, streptomycin biosynthesis, Vitamin B6, butanoate, glycine, serine, and threonine metabolism showed significant upregulation in smokers compared to nonsmokers. While thiamine metabolism, D-glutamine and D-glutamate metabolism, D-Aarginine and D-ornithine metabolism, homologous recombination, epithelial cell signaling in helicobacter pylori, Aminoacyl-tRNA biosynthesis, phosphonate and phosphinate metabolism, polycyclic aromatic hydrocarbon degradation, synthesis and degradation of ketone bodies, translation factors, Ascorbate/aldarate metabolism, and glycosaminoglycan degradation DNA replication showed significant upregulation in nonsmokers (Figure 2(c)).

### 3.5. Metagenome Predictions between Nonsmokers and Smokers-CP Granulation Tissue Samples and Associated Functions

**Figure 3:**
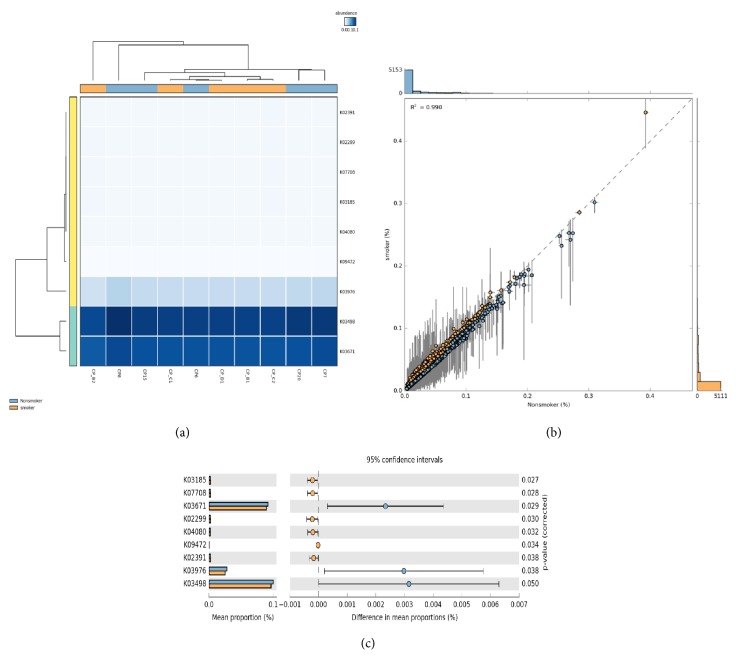
(a)** Heatmap profile of metagenome predictions** of identified species between nonsmokers and smokers in granulation tissue of chronic periodontitis samples. (b) Scatter plot and correlation estimates of bacterial species functional predictions between nonsmokers and smokers in granulation tissue of chronic periodontitis samples. (c) Mean proportion of bacterial species functional predictions between nonsmokers and smokers in granulation tissue of chronic periodontitis samples. The significant differences observed between the two groups at 95% confidence level and p>0.05 are reported.

Metagenome predictions of the observed species abundance showed the formation of no distinct clusters in heatmap profiles (Figure 3(a)), with a correlation coefficient of R^2^ = 0.990 (Figure 3(b)). Although two distinct clusters could be identified, where one cluster included observational ID's K03671 (Thioredoxin 1), K03498 (trk system potassium uptake protein TrkH), and K03976 (putative transcription regulator) and other cluster comprised K02299 (cytochrome o ubiquinol oxidase subunit III [EC:1.10.3.-]), K02391 (flagellar basal-body rod protein FlgF), K03185 (2-octaprenyl-6-methoxyphenol hydroxylase [EC:1.14.13.-]), K04080 (molecular chaperone IbpA), K07708 (two-component system, NtrC family, nitrogen regulation sensor histidine kinase GlnL (EC:2.7.13.3)), K09472 (gamma-glutamyl-gamma-aminobutyraldehyde dehydrogenase (EC:1.2.1.-). Extended error plot showed significant (p>0.05) upregulation of observational IDs K03671 (Thioredoxin 1), K03498 (trk system potassium uptake protein TrkH), and K03976 (putative transcription regulator) in nonsmokers and second cluster IDs showed significant (p>0.05) upregulation in smokers (Figure 3(c)).

## 4. Discussion

In the present study a total of 8 assigned phyla among which dominating were* Firmicutes *and* Bacteroidetes *comprising more than >40% species, while less than <40% were represented by* Fusobacteria, Actinobacteria*,* Spirochaetes, Tenericutes*, and* Chloroflexi *across control and experimental samples. In an earlier study, Kumar et al. [32] showed the presence of* Firmicutes*,* Proteobacteria*,* Actinobacteria*,* Bacteroidetes*,* Spirochaetes*, and* Synergistes *in samples collected from the margin and subgingival plaques tissue. Although the species* Selenomonas noxia, Selenomonas bovis*, and* Veillonella parvula *in phyla-Firmicutes*; Neisseria oralis *and* Campylobacter rectus *in Phyla Proteobacteria*; Capnocytophaga ochracea*,* Macellibacteroides fermentans*, and* Prevotella nigrescens *in phyla* Bacteroidetes *showed equivalent abundance as earlier reported [10]. Recently,* Veillonella *spp.,* Streptococcus *spp.,* Prevotella *spp., and* Lactobacillus *spp. were reported predominantly to be abundant among children's with caries [33]. However, differential abundance analysis through PICRUST analysis after normalization of OTU's showed the dominance of Phyla-*Fusobacteria*-*Streptobacillus moniliformis*; Phyla-*Firmicutes*-*Streptococcus equi*; Phyla* Proteobacteria*-*Enhydrobacter aerosaccus*; Phyla-*Firmicutes*-*Staphylococcus saprophyticus *in nonsmokers (p>0.05) compared to smokers. This differential dominance of these species among nonsmokers led significant minor upregulation of KEGG pathways involved in primary immunodeficiency which may increase the susceptibility to infections in smokers as supported by Ballow et al. [34] that primary immunodeficiency is uncommon, chronic, and severe disorders of the immune system in which patients cannot mount a sufficiently protective immune response, leading to an increased susceptibility to infections. We also observed upregulation of citrate cycle, metabolism of Vitamin B6, butanoate, glycine, serine and threonine in smokers which has also been shown by Barupal et al., [35], where they reported that environmental tobacco smoke (ETS) exposure in adult rats shows adverse effects on the mitochondrial respiratory chain, lung elasticity, membrane integrity, redox states, cell cycle, and normal metabolic and physiological functions of lungs, even after subchronic ETS exposure. Further, alteration in metabolism of thiamine, D-glutamine and D-glutamate, D-arginine, and D-ornithine has also been earlier reported [36] and inhibition of pancreatic acinar mitochondrial thiamin pyrophosphate uptake by the cigarette smoke component 4-(methylnitrosamino)-1-(3-pyridyl)-1-butanone was suggested. Vulimiri et al. [37] showed that global metabolome is affected by whole smoke significantly and most importantly alters glutathione (GSH levels), glutamine and increased polyamine, and pantothenate (vitamin B5) phospholipid degradation.

A decrease in homologous recombination and DNA replication showed trivial significant downregulation in smokers suggestive of higher formation of DNA adducts, which may lead to the process of carcinogenesis. Decrease in aminoacyl-tRNA biosynthesis and translation factors is supported by the recent evidence that suggests that RNA species are required at initiation, because treatment of cells with antibiotics, drugs, or chemical carcinogens (hydrocarbons of smoking) may inhibit RNA synthesis (including both rRNA and tRNA) and cause a decrease in protein synthesis [38]. A decrease in synthesis and degradation of ketone bodies, which are particularly important for the brain which has no other substantial nonglucose-derived energy source, may further alter both the tricarboxylic acid cycle and oxidative phosphorylation pathways and may lead to oxidative stress [39]. Additionally, a decrease in phosphonate and phosphinate metabolism, polycyclic aromatic hydrocarbon degradation, ascorbate and aldarate metabolism and glycosaminoglycan degradation, and glycosyltransferases may further modulate the metabolism and hence niche of the granulation tissue.

A high abundance of bacterial species associated with K09472-gamma-glutamyl-gamma-aminobutyraldehyde dehydrogenase was involved in oxidative stress; K04080-molecular chaperone IbpA was involved in cell division and cell cycle; K03185 2-octaprenyl-6-methoxyphenol hydroxylase (EC:1.14.13.-) was involved in cofactors, vitamins, prosthetic groups, pigments, quinone cofactors, and ubiquinone biosynthesis; and K02299 cytochrome O ubiquinol oxidase subunit III (EC:1.10.3.-) was involved in respiration and electron accepting reactions may be associated with the severity of chronic periodontitis in smokers.

## 5. Conclusions

In the present work, we demonstrated the diversity and complexity of the oral microbial community associated with chronic periodontitis and species richness affected by smoking. At the species level, dysbiosis was caused due to smoking complicating the disease conditions modulating the global metabolic pathways related to amino acids, vitamins, phosphonate and phosphinate, ascorbate and aldarate metabolism. In addition, increase in primary immunodeficiency; decrease in homologous recombination and DNA replication; decrease in degradation of ketone bodies and polyaromatic hydrocarbons pathways in smokers may lead to the increased risk of carcinogenesis.

## Figures and Tables

**(a):** 

**Region**		**Forward Primers (with forward barcode)**	**Amplicon length (Without Barcode)**
V1-V9	F	AGRGTTYGATYMTGGCTCAG	1,464
	F1	TGAGTGACGTGTAGCGAGRGTTYGATYMTGGCTCAG	
	F2	GACAGCATCTGCGCTCAGRGTTYGATYMTGGCTCAG	
	F3	TGCGAGCGACTCTATCAGRGTTYGATYMTGGCTCAG	
	F4	TGCTCTCGTGTACTGTAGRGTTYGATYMTGGCTCAG	

**(b):** 

**Region**		**Reverse Primers (with reverse barcode)**	**Amplicon length (Without Barcode)**
V1-V9	R	RGYTACCTTGTTACGACTT	1,464
	R1	GCTCGACTGTGAGAGARGYTACCTTGTTACGACTT	
	R2	TGCTCGCAGTATCACARGYTACCTTGTTACGACTT	
	R3	GCAGACTCTCACACGCRGYTACCTTGTTACGACTT	

	R4	AGACAGCATCTGCGCTCRGYTACCTTGTTACGACTT	

**Table 2:** **Determination of species/phyla/genus in the control (CP) and experimental (CP+S) granulation tissue samples using PacBio sequencing:** the observed number of OTUs based on high-quality sequences processed through Mothur package against green gene_13_8_99.gg.tax database for species richness in pooled 5 control (Chronic Periodontitis) and 5 experimental (chronic periodontitis + smoking) samples.

	CP		CP+S	
Proteobacteria	15224	11.85531	52312	13.0633

Firmicutes	74333	57.88498	211196	52.73967

Bacteroidetes	28475	22.1742	100092	24.99488

Actinobacteria	2609	2.031694	9343	2.333125

Fusobacteria	6077	4.732313	21842	5.454364

Spirochaetes	1642	1.278667	5469	1.365714

Tenericutes	2	0.001557	3	0.000749

Chloroflexi	53	0.041272	193	0.048196

	128415		400450	

## Data Availability

The data used to support the findings of this study are available from the corresponding author upon request.

## Supplementary Materials

Supplementary Materials
** Supplementary Figure 1: taxonomic hits distribution of five (CP+S) patients' sample:** the charts represent the distribution of taxa using a contigLCA algorithm finding a single consensus taxonomic entity at phylum level on each individual sample.** Supplementary Figure 2: taxonomic hits distribution of five (CP+S) patients' sample:** the charts represent the distribution of taxa using a contigLCA algorithm finding a single consensus taxonomic entity at genus level on each individual sample.** Supplementary Figure 3: taxonomic hits distribution of five (S) control sample:** the charts represent the distribution of taxa using a contigLCA algorithm finding a single consensus taxonomic entity at phylum level on each individual sample.** Supplementary Figure 4: taxonomic hits distribution of five (S) control sample:** the charts represent the distribution of taxa using a contigLCA algorithm finding a single consensus taxonomic entity at genus level on each individual sample.** Supplementary Figure 5: alpha diversity of five control (a) and experimental (b) samples:** the above images show the range of *α*-diversity values. The min, max, and mean values are shown, with the standard deviation ranges (*σ* and 2*σ*) in different shades. The *α*-diversity of this metagenome is shown in red.** Supplementary Figure 6: rarefaction curve of five control (a) and experimental (b) samples:** the plot shows the rarefaction curve of the total number of annotated species richness per sample as a function of the number of sequences sampled.** Supplementary Table 1:** screening of 30 chronic periodontitis (CP) patients where five patients (CP_B1, CP_B2, CP_C1, CP_C2, and CP_D1) with the history of smoking and tobacco chewing, bleeding on probing, and periodontal pockets ≥ 5mm and having clinical attachment loss (CAL) were selected as experimental group (CP+S). Five patients of corresponding age group (CP_6, CP_7, CP_8, CP_15, and CP_20) with the history of no smoking and tobacco chewing, bleeding on probing, and periodontal pockets ≥ 5mm and having clinical attachment loss (CAL) were included as control group. Raw reads and filtered consensus sequences (CCS) were obtained after demultiplexing of barcoded individual samples.
