# Supplementary material for: 16S rRNA Long-Read Sequencing of the Granulation Tissue from Nonsmokers and Smokers-Severe Chronic Periodontitis Patients
Source: Biomed Res Int. 2018 Jun 14;2018:4832912. doi: 10.1155/2018/4832912 (PMC6022309; doi:10.1155/2018/4832912)
Supplement: Supplementary Materials — Supplementary Figure 1: taxonomic hits distribution of five (CP+S) patients' sample: the charts represent the distribution of taxa using a contigLCA algorithm finding a single consensus taxonomic entity at phylum level on each individual sample. Supplementary Figure 2: taxonomic hits distribution of five (CP+S) patients' sample: the charts represent the distribution of taxa using a contigLCA algorithm finding a single consensus taxonomic entity at genus level on each individual sample. Supplementary Figure 3: taxonomic hits distribution of five (S) control sample: the charts represent the distribution of taxa using a contigLCA algorithm finding a single consensus taxonomic entity at phylum level on each individual sample. Supplementary Figure 4: taxonomic hits distribution of five (S) control sample: the charts represent the distribution of taxa using a contigLCA algorithm finding a single consensus taxonomic entity at genus level on each individual sample. Supplementary Figure 5: alpha diversity of five control (a) and experimental (b) samples: the above images show the range of α-diversity values. The min, max, and mean values are shown, with the standard deviation ranges (σ and 2σ) in different shades. The α-diversity of this metagenome is shown in red. Supplementary Figure 6: rarefaction curve of five control (a) and experimental (b) samples: the plot shows the rarefaction curve of the total number of annotated species richness per sample as a function of the number of sequences sampled. Supplementary Table 1: screening of 30 chronic periodontitis (CP) patients where five patients (CP_B1, CP_B2, CP_C1, CP_C2, and CP_D1) with the history of smoking and tobacco chewing, bleeding on probing, and periodontal pockets ≥ 5mm and having clinical attachment loss (CAL) were selected as experimental group (CP+S). Five patients of corresponding age group (CP_6, CP_7, CP_8, CP_15, and CP_20) with the history of no smoking and tobacco chewing, bleeding on probing, and periodo [file 4832912.f1.pdf]

Supplementary Figure 1: Taxonomic Hits Distribution of Five (CP+S) patients sample: The charts represent the distribution of taxa using a contigLCA algorithm finding a single consensus taxonomic entity at Phylum level on each individual sample.

Supplementary Figure 2: Taxonomic Hits Distribution of Five (CP+S) patients sample: The charts represent the distribution of taxa using a contigLCA algorithm finding a single consensus taxonomic entity at genus level on each individual sample.

Supplementary Figure 3: Taxonomic Hits Distribution of Five (S) Control sample: The charts represent the distribution of taxa using a contigLCA algorithm finding a single consensus taxonomic entity at Phylum level on each individual sample.

Supplementary Figure 4: Taxonomic Hits Distribution of Five (S) Control sample: The charts represent the distribution of taxa using a contigLCA algorithm finding a single consensus taxonomic entity at genus level on each individual sample.

Supplementary Figure 5: Alpha diversity of five control (a) and experimental (b) samples: The above images show the range of α-diversity values. The min, max, and mean values are shown, with the standard deviation ranges (σ and 2σ) in different shades. The α-diversity of this metagenome is shown in red.

Supplementary Figure 6: Rarefaction curve of five control (a) and experimental (b) samples: The plot shows the rarefaction curve of the total number of annotated species richness per sample as a function of the number of sequences sampled.

Supplementary Table 1: Screening of 30 Chronic Periodontitis (CP) patients where, five patients (CP_B1, CP_B2, CP_C1, CP_C2, CP_D1) with the history of smoking and tobacco chewing, bleeding on probing, Periodontal pockets ≥ 5mm and having Clinical attachment loss (CAL) were selected as experimental group (CP+S). Five patients of corresponding age group (CP_6, CP_7, CP_8, CP_15, CP_20) with the history of no smoking and tobacco chewing, bleeding on probing, Periodontal pockets ≥ 5mm and having Clinical attachment loss (CAL) were included as control group. Raw reads and filtered consensus sequences (CCS) obtained after demultiplexing of barcoded individual samples.

**Group**	**Sample**	**Age**	**Raw Reads**	**Filtered Reads**
Control (CP without smoking)	CP6	50	18441	12727
	CP7	60	22446	18378
	CP8	60	14842	7825
	CP15	55	14778	10887
	CP20	66	11332	5860
Experimental (CP with Smoking)	CP_D1	60	12658	1379
	CP_B1	42	21749	19564
	CP_B2	50	15976	10155
	CP_C1	47	14342	5197
	CP_C2	67	16982	7194
Total Reads			163546	99166

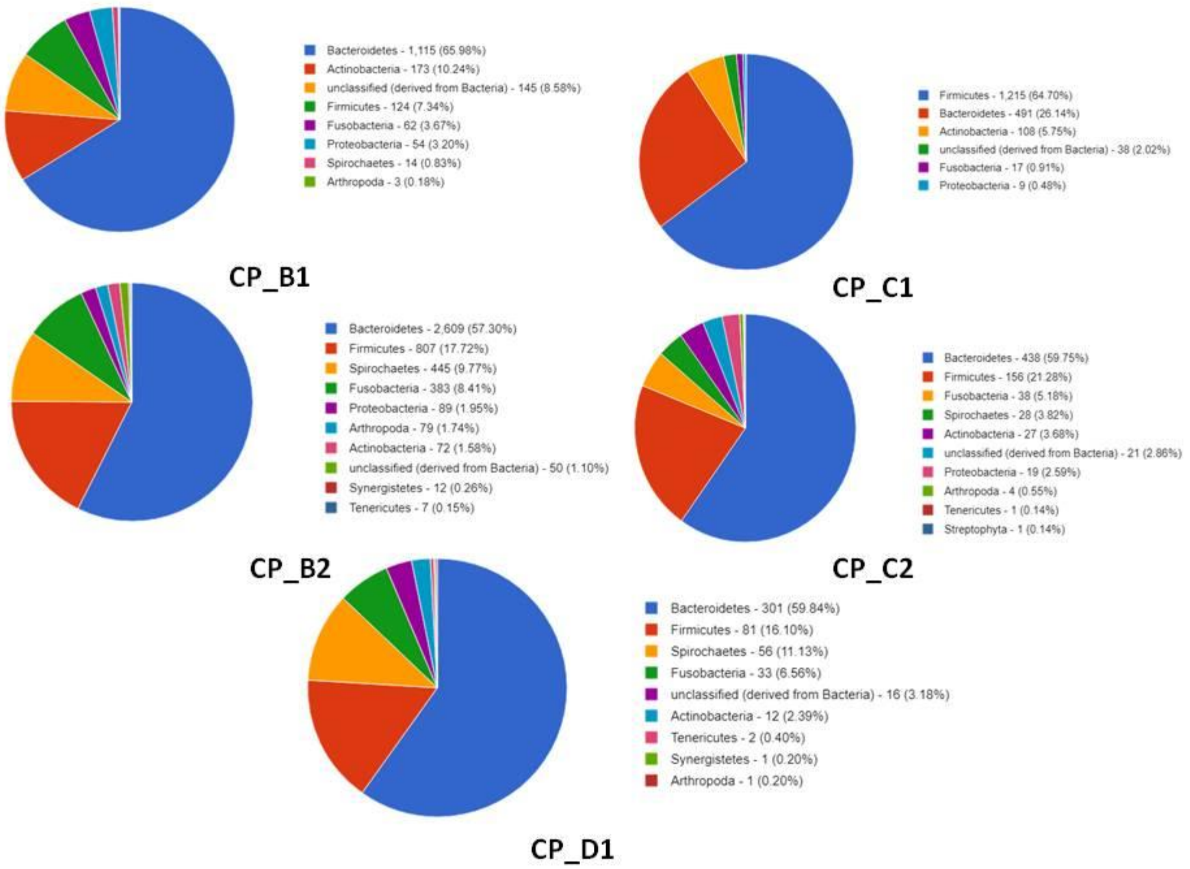

Supplementary Figure 1

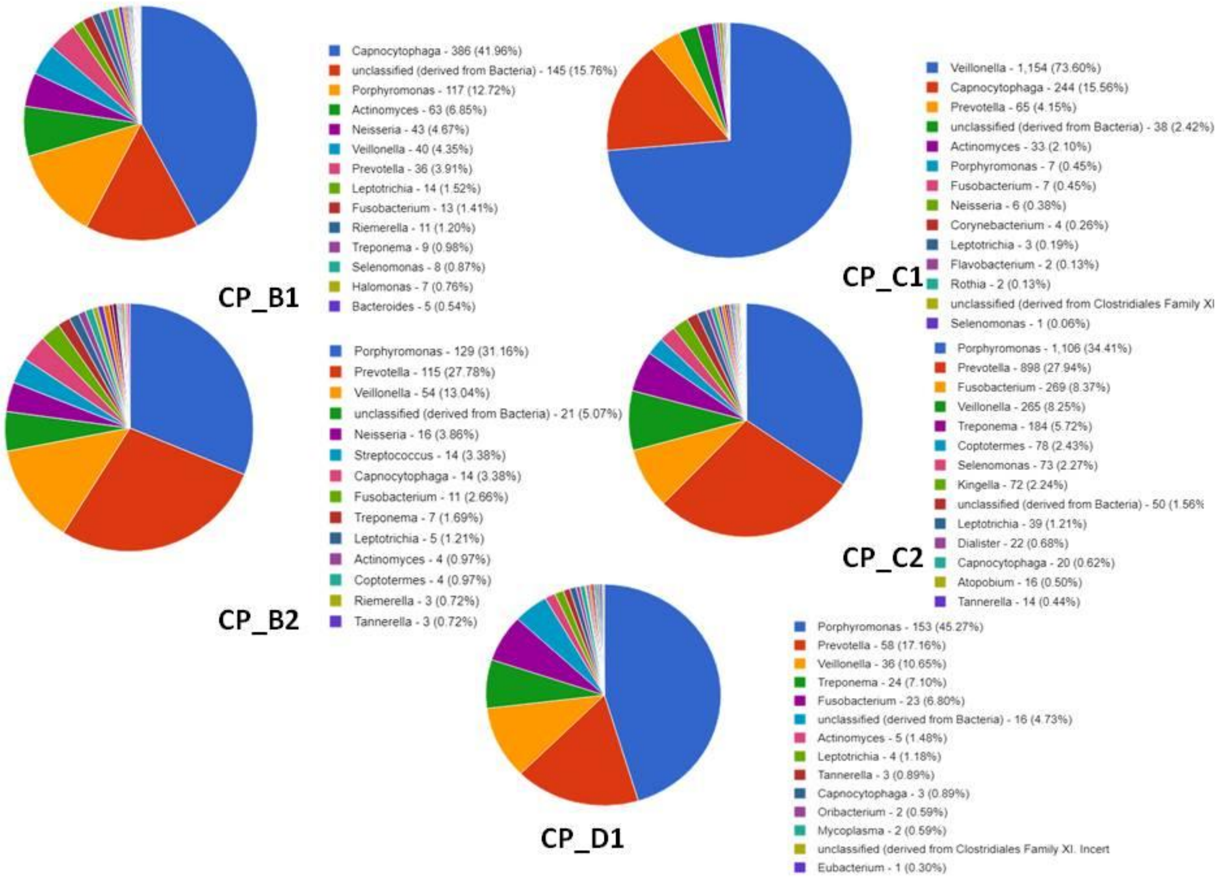

Supplementary Figure 2

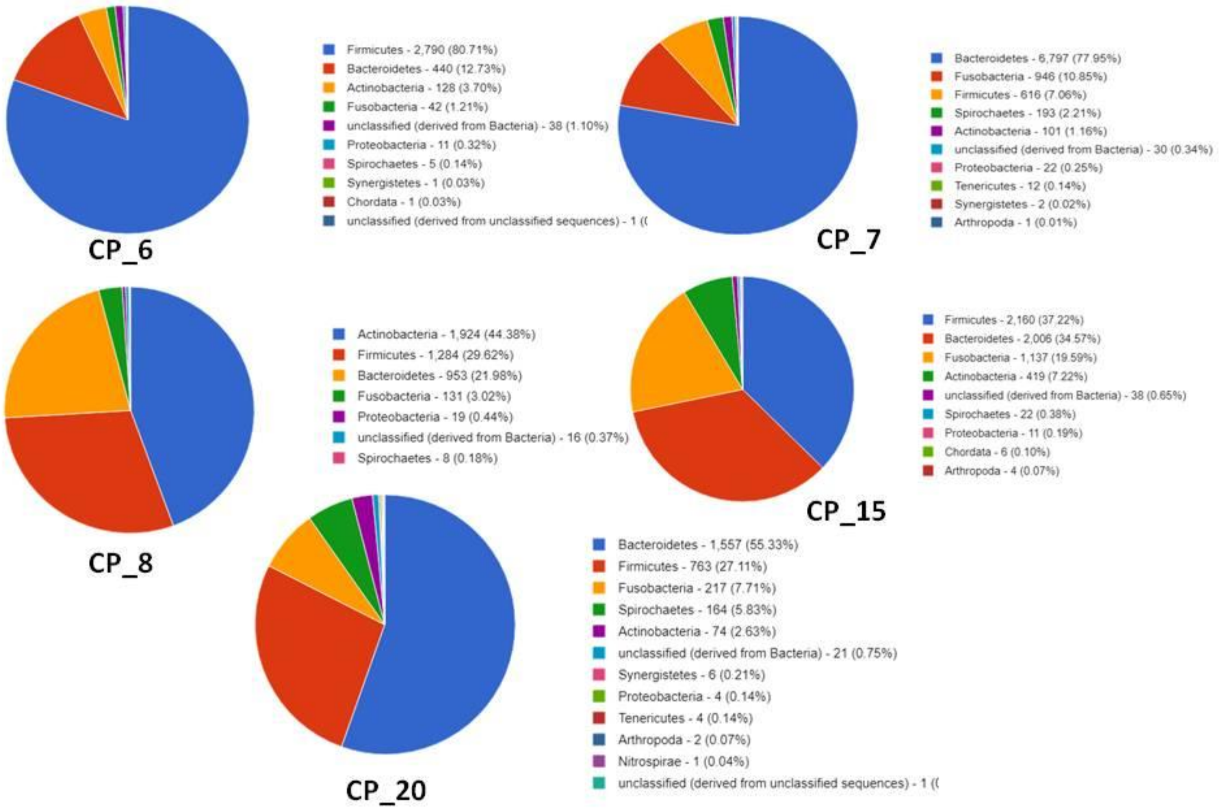

Supplementary Figure 3

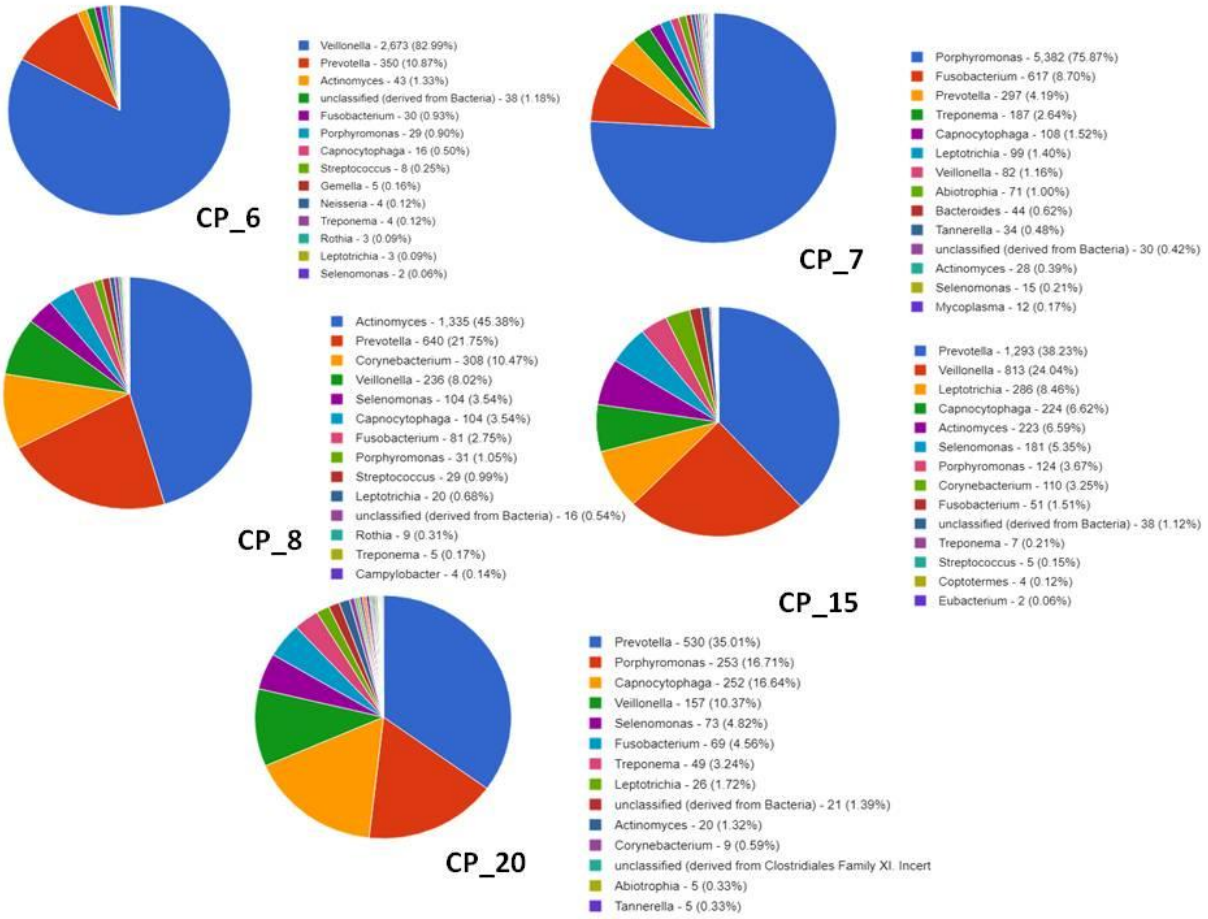

Supplementary Figure 4

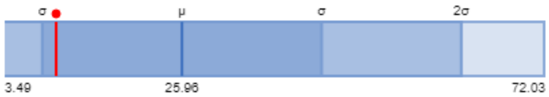

CP-6: The α-diversity of this data set is 10 species.

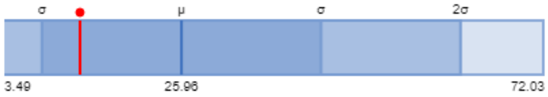

CP_7: The α-diversity of this data set is 13 species.

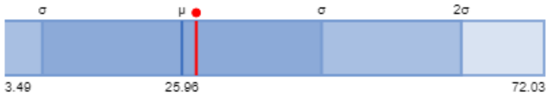

CP-8: The α-diversity of this data set is 28 species.

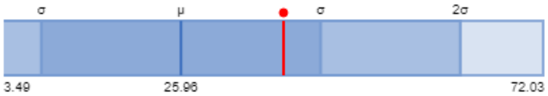

CP_15: The α-diversity of this data set is 39 species.

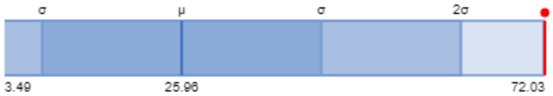

CP-20: The α-diversity of this data set is 72 species.

(a)

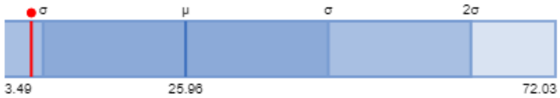

CP-B1: The α-diversity of this data set is 7 species.

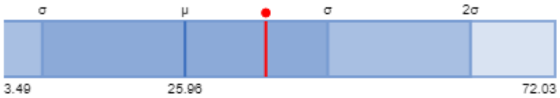

CP_B2: The α-diversity of this data set is 36 species.

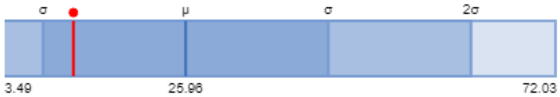

CP_C1: The α-diversity of this data set is 12 species.

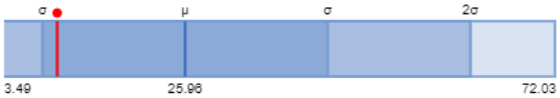

CP_C2: The α-diversity of this data set is 10 species.

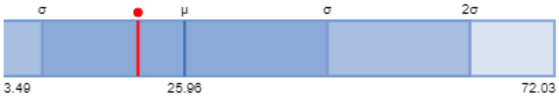

CP_D1: The α-diversity of this data set is 20 species.

(b)

Supplementary Figure 5

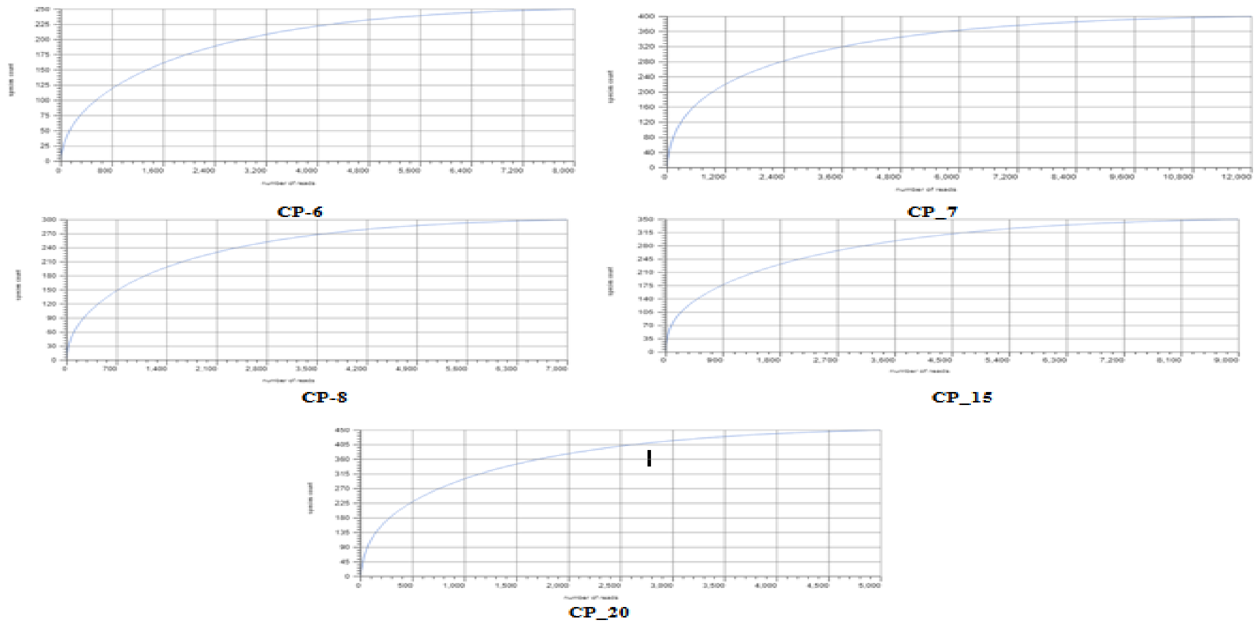

(a)

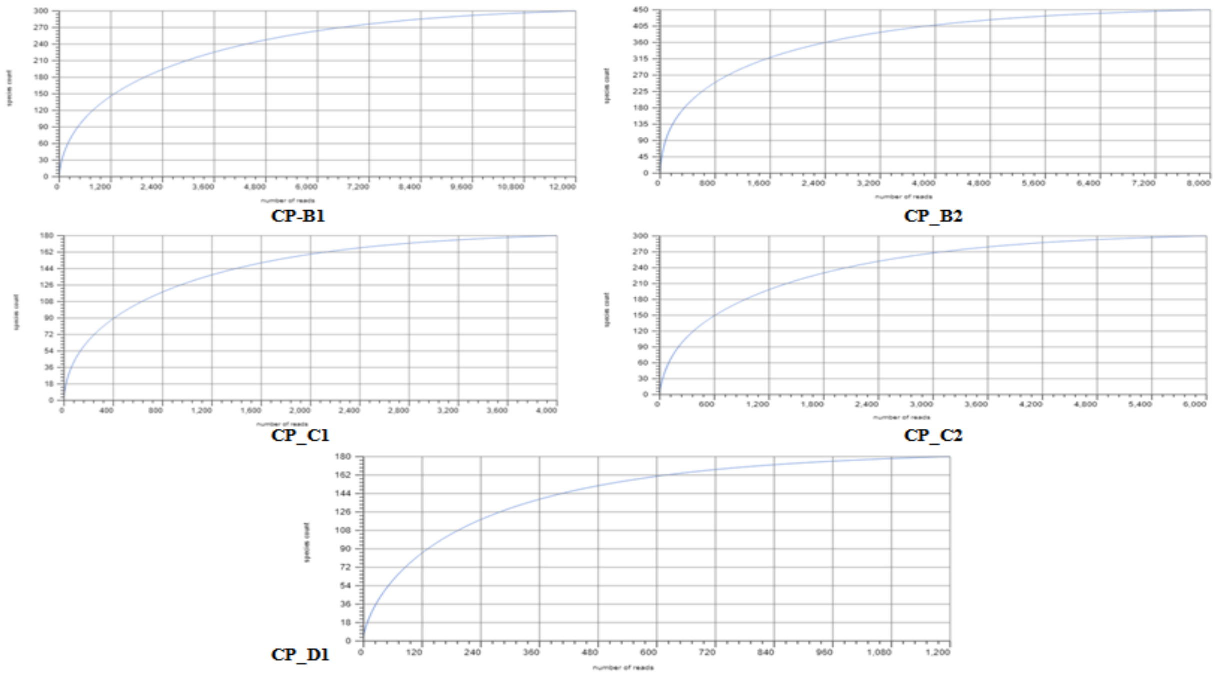

(b)

Supplementary Figure 6
